# Antibacterial and Cytotoxic Potential of Biosynthesized Silver Nanoparticles by Some Plant Extracts

**DOI:** 10.3390/nano8060382

**Published:** 2018-05-30

**Authors:** Afrah E. Mohammed, Alaa Al-Qahtani, Amal al-Mutairi, Bashayir Al-Shamri, Kawther Aabed

**Affiliations:** Department of Biology, Faculty of Sciences, Princess Nourah Bint Abdulrahman University, 11474 Riyadh, Saudi Arabia; aaxx0013@hotmail.com (A.A.-Q.); Amlhadi-@hotmail.com (A.a.-M.); besh053@hotmail.com (B.A.-S.); dr.kaabed@gmail.com (K.F.A.)

**Keywords:** AgNPs, *Phoenix dactylifera*, *Acacia nilotica*, *Ferula asafoetida*, antibacterial, cytotoxicity

## Abstract

The provision of nanoparticles using biogenic material as a part of green chemistry is an attractive nanotechnology. The current research aimed to test the antimicrobial and cytotoxic efficacy of silver nanoparticles synthesized by extracts of *Phoenix dactylifera*, *Ferula asafetida*, and *Acacia nilotica* as reductant and stabilizing agents in silver nanoparticle formation. Synthesized nanoparticles were evaluated for their antimicrobial activity against *Staphylococcus aureus* (Gram-positive) and *Pseudomonas aeruginosa* and *Escherichia coli* (Gram-negative) using an agar well diffusion assay. Furthermore, cytotoxic ability was investigated against LoVo cells. The potential phyto-constituents of plant extracts were identified by Fourier-transform infrared spectroscopy (FT-IR) techniques. Field emission scanning electron microscopy (FE-SEM), transmission electron microscope (TEM), and zeta potential analyzed the size and morphology of the biogenic nanoparticles. The current study revealed the ability of the tested plant extract to convert silver ions to silver nanoparticles with an average size that ranged between 67.8 ± 0.3 and 155.7 ± 1.5 nm in diameter. Biogenic AgNPs showed significant antibacterial ability (10 to 32 mm diameter) and anticancer ability against a LoVo cell with IC50 ranged between 35.15–56.73 μg/mL. The innovation of the present study is that the green synthesis of NPs, which is simple and cost effective, provides stable nano-materials and can be an alternative for the large-scale synthesis of silver nanoparticles.

## 1. Introduction

Recently, worldwide problems associated with environmental concerns have led to a focus on the technology of green chemistry as an environmentally friendly process in chemistry to overcome different health problems [1]. The development of antibiotic resistant bacteria is highly associated with an increase in antibiotic usage and is one of the recent global health problems. Consequently, great efforts are being made to look for alternatives to antibiotics to stop the development of antibiotic resistant microbes. Silver is one of the metal ions that has shown potential bactericidal, anti-fungal, and anti-inflammatory effects [2,3]. Furthermore, silver in the form of nanoparticles (AgNPs) has been used against microorganisms and exhibited a promising way to resolve the development of antibiotic-resistant bacteria [4]. AgNPs and their role as cytotoxic materials against cancer cells was reported in [5,6]. The conversion of silver ion to silver nanoparticles using chemical reduction, ion sputtering, and sol gel are well known techniques [7,8,9,10], but such practices may have a negative environmental influence since hazardous chemicals might be included in such a conversion; therefore, looking for environmentally friendly alternatives is necessary. A green synthesis of nanoparticles using biological material showed an advantage over other methods since it is simple, cost effective, and produces a stable product [11]. Plant extracts and microorganisms can be utilized to convert metal ion to metal nanoparticles as an alternative to conventional methods. Recent studies used different plant extracts as a mediator for silver nanoparticle formation such as *Eucalyptus camaldulensis*, *Ziziphus spina christi*, *Calligonum comosum*, marigold flower and *Ziziphora tenuior*, *Azadirachta indica* [7,9,12,13,14,15,16], and *Erythrina indica* [10]. On the other hand, different microbes were also studied as the bio-mediator in silver nanoparticle formation such as *E. coli* [17], a cell-free supernatant derived from *Bacillus* sp. culture [18], *Aspergillus flavus*, *Lactic acid bacteria*, *Bacillus licheniformis*, and *Bacillus cereus*, [19,20,21,22], but plant extracts showed a higher ability for the conversion of Ag ions to AgNPs compared to conversion mediated by microorganisms [23]. This might be due to the fact that plants are a rich source of biologically active compounds such as flavones, ketones, aldehydes, amides, carboxylic acids, proteins, DNA, and enzymes. Such compounds act as bio-reducing agents and mediate the reduction process of Ag ions to AgNPs [24]. On the other hand, the antimicrobial ability of AgNPs against a wide spectrum of MDR pathogens was largely investigated [25,26,27]. A recent study suggested the mechanism of AgNPs on bacterial cell death is its influence on membrane morphology that leads to improper permeability and material transport [28]. Plant extracts and their high potentiality in AgNPs biosynthesis, beside their antibacterial and cytotoxicity, were the aims of the current investigation since the plant is a rich source of phytochemicals. In this context, the extract of date palm *Phoenix dactylifera* L. is studied. Date palm is one of the fruit trees in the Arab region that it is widely grown and has edible sweet fruit. Recently, different studies investigated the ability of the *Phoenix dactylifera* fruit and plant leaves aqueous extract in the synthesis of AgNPs and palladium nanoparticles [29,30,31]. Ajwa, a type of date that is only cultivated in Al-Madinah Al-Munawara/Saudi Arabia, is investigated. Ajwa showed a high free radical scavenging ability via its antioxidant properties and a high content of poly phenol with a highly significant impact on disease cures in different studies [32,33]. On the other hand, *Acacia nilotica*, is a medicinal plant that has a high antioxidant capability [34]. *A. nilotica* gum extract was recently used as the biomediator in AgNPs formation [35]. Furthermore, a plant of the *Ferula asafoetida* L. variety is common in Iran and a main source of asafetida, which is produced by the plant as the root exudates [36]. As a result, it is one of the target plants in the current study. Synthesized AgNPs using *Phoenix dactylifer*, *Acacia nilotica*, and *Ferula asafoetida* were detected by a scanning electron microscope (SEM), transmission electron microscope (TEM), and zeta potential. Furthermore, the bactericidal activity of AgNPs was tested against three human pathogenic bacteria for minimum inhibitory concentration (MIC) determination. The cytotoxic effect was also determined against LoVo cell lines by 3-(4,5-dimethyl-2-thiazolyl)-2,5-diphenyl-tetrazolium bromide (MTT) assay.

## 2. Materials and Methods

### 2.1. Collection and Storage of Plant Samples

The fruit samples of *Acacia nilotica* and *Ferula asafoetida* were collected from the Riyadh region in Saudi Arabia, and *Phoenix dactylifera* L. (Ajwah) was collected from the Almadinah Almunawwarah region in Saudi Arabia. Samples were labelled and stored at 4 °C in polythene bags for further processing. The plant parts were washed with distilled water and dried. Dried samples were ground well into a fine powder with the help of a milling machine (IKA werke, GMBH and Co., Staufen im Breisgau, Germany). The powder was stored in air sealed plastic containers at room temperature for extraction and further analysis.

### 2.2. Synthesis of Silver Nanoparticles (AgNPs)

Aqueous and ethanolic extracts were prepared from the collected plant materials by adding 10 g powder to 100 mL solvent. Heat treatment for 10 min at 80 °C to stop the enzymes activity was performed on the aqueous extract. The solution then filtered through whatman candidate No. 1 (pore size 125 mm, Maidstone, England,). Furthermore, the ethanolic extract was kept overnight and then filtered through the same whatman candidate mentioned above. Filtrate was heated for the concentration of the extract and then kept for further use. For the synthesis of the AgNPs, 10 mL of each prepared extract as reducing and capping agents were mixed with 90 mL of a 1 mM AgNO_3_ solution in an Erlenmeyer flask and allowed to react at room temperature for 48 h. For each sample, preparation was done three times for repeatability. AgNPs were stored for further study at temperature of 4 °C.

### 2.3. Characterization of Biogenic AgNPs

UV Spectroscopy, Dynamic light scattering, zeta potential, field emission scanning electron microscopy (Peabody, MA, USA), and transmission electron microscopy (Peabody, MA, USA) were used for detection of biogenic AgNPs as follows:

#### 2.3.1. UV Spectroscopy

UV-visible spectrophotometer (Shimadzu, Tokyo, Japan) was used for the characterization of AgNPs. The reduction of pure Ag^+^ ions was checked by measuring at UV-2450 double-beam (200–800 nm).

#### 2.3.2. Dynamic Light Scattering (DLS) and Zeta Potential

A Zetasizer nano device (Malvern, Worcestershire, UK) was used to measure the size and distribution of AgNPs prepared by plant extracts.

#### 2.3.3. Field Emission Scanning Electron Microscopy (FE-SEM)

The FE-SEM technique (JEOL 7500FA JEOL, Peabody, MA, USA) was used to reveal information about the sample, including the external morphology materials making up the samples. A drop of nanoparticle suspensions with a volume of 8 μL was placed onto 200 mesh grids with a carbon support film (Agar Scientific, London, UK) and dried. Furthermore, the sample was rinsed with EtOH, dried and fixed on an appropriate SEM holder. Images were taken at an accelerating voltage of 30 kV. JEOL JEM-2100 (JEOL, Peabody, MA, USA).

#### 2.3.4. Transmission Electron Microscopy (TEM)

TEM a JEOL JEM-1011 (JEOL, Peabody, MA, USA) was used for size, shape, and crystallinity characterization of the biogenic AgNPs. Samples were prepared by placing a small drop with a volume of 8 μL on a carbon coated copper grid of 300 meshes. Images were taken at an acceleration voltage of 200 kV.

#### 2.3.5. Fourier-Transform Infrared Spectroscopy (FT-IR)

FT-IR is a method of measuring infrared absorption and emission spectra of the biomolecules found in the prepared samples. A range of 500–4000 cm^−1^ FT-IR (Nicolet 6700 FT-IR Spectrometer, Waltham, MA, USA) was used.

### 2.4. Evaluation of the Antibacterial Activity of AgNPs

The antibacterial activity of AgNPs was determined using well agar diffusion methods [14]. Three types of bacteria, *Staphylococcus aureus* from the Gram-positive group and *Pseudomonas aeruginosa* and *Escherichia coli* from the Gram-negative group, were tested. Pure cultures of the microorganisms were sub-cultured on Mueller-Hinton Agar. 0.2 mL of bacteria strain (2.5 × 10^5^ CFU/mL) was swabbed uniformly onto individual agar plates using sterile swabs. Subsequently, three adequately spaced wells (holes), each 4 mm in diameter, were made per plate at the culture agar surface using a sterile metal cork borer. In each hole, 0.2 mL of extract was used under aseptic conditions, kept at room temperature for one hour to allow the extracts to diffuse into agar medium and incubated accordingly. Sterile distilled water was used as the reference negative control. Plates were incubated at 37 °C for 18–24 h. Inhibition zones that appeared as a clear area around the wells were evaluated.

#### 2.4.1. Minimum Inhibitory Concentration (MIC) and Minimum Bactericidal Concentration (MBC) Determination

The MIC and MBC values were determined by a micro dilution method in NB. 10 µL of bacterial strain containing 2.5 × 10^5^ CFU/mL, bacteria was added individually to 10 mL of NB. Different concentrations of AgNPs were added to the test tubes containing the bacterial strains and incubated for 24 h. After incubation, the MIC values were obtained by checking the turbidity of the bacterial growth. The MIC value corresponded to the concentration that inhibited 99% of bacterial growth. Furthermore, the lowest concentration of AgNPs that completely killed the tested bacteria was considered as the MBC [28].

#### 2.4.2. Tolerance Level

The tolerance levels against AgNPs were determined for each bacterial strain using the following formula [37].

Tolerance = MBC/MIC

The bactericidal capacity of the analyzed compound was detected by the tolerance level reflecting the bactericidal and bacteriostatic agents against tested microbes. If the ratio is ≥16, then the agent has a bacteriostatic effect whereas the ratio ≤4 reflects the bactericidal ability of the examined material [38].

On the other hand, to study the interaction of biogenic AgNPs with tested microbes, *bacillus* sp. was assessed using FE-SEM. Broth containing *Pseudomonas aeruginosa* exposed to AgNPs was subjected to SEM after 24 h.

#### 2.4.3. Synergistic Antibacterial Potential Silver Nanoparticles

The synergistic antibacterial effect of AgNPs and Amoxicillin, Ciprofloxacin and Cefuroxime as standard antibiotics was tested against *S. aureus*, *P. aeruginosa*, and *E. coli* using a disk diffusion method (Naqvi et al., 2013). Fresh cultured bacteria were used to study the synergistic effect. The antibiotic disks were mixed by 1 mL of AgNPs and placed at the plates cultured with bacteria. The synergistic antibacterial activity of AgNPs and antibiotics combination was measured after 24 h of incubation at 37 °C as diameter of inhibition zone around the disks (mm).

### 2.5. Cancer Cell Lines and Culture Conditions

LoVo cells lines were obtained from the Faculty of Science, Kind Saud University, Riyadh, Saudi Arabia. The cytotoxic potency of AgNPs mediated by plant extract was tested against LoVo cell lines by the MTT test, which depends on the reduction of MTT tetrazolium salt to purple formazan due to the presence of metabolically active cells [39]. A Beckman Coulter spectrophotometer (USA) was used for the MTT test to estimate the proliferation rates at 595 nm. The viable cell percentage was calculated, taking into account the 100% viability of untreated cells. 50% of the Inhibitory Concentration (IC50) was used for the cytomorphological observation. After the treatment, the cells (control and treated) were washed with PBS and fixed at 1:1 ratio of methanol and glacial acetic acid for 1 h at room temperature.

$$\mathrm{Viability}\;\%=\frac{\mathrm{OD}\;\mathrm{sample}}{\mathrm{OD}\;\mathrm{control}}\times \;100$$

## 3. Statistical Analysis

Each test in the present investigation was performed at least three times for repetitions; means and standard deviations were calculated using Microsoft Excel 2013. The SEM and TEM images of the AgNPs were chosen from one of the repetitions.

## 4. Results and Discussion

### 4.1. AgNPs Synthesis Using Plant Extracts and AgNPs Characterization

**Figure 1:**
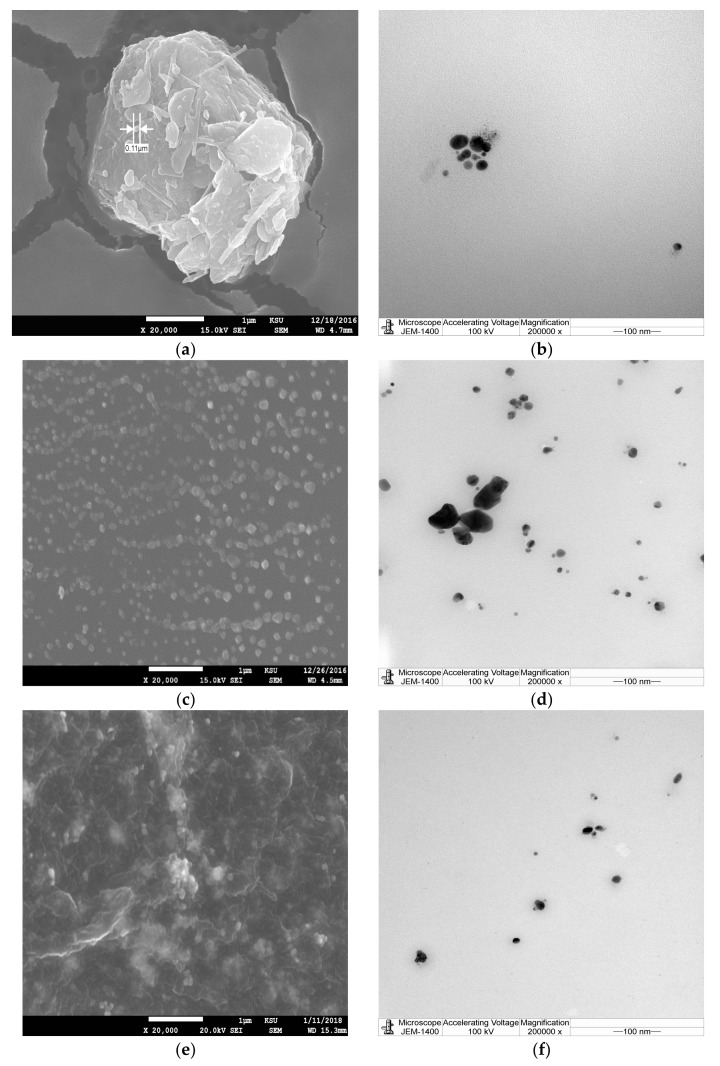
Scanning electron microscope (SEM) images (**a**–**c**) and the corresponding transmission electron microscopy (TEM) images (**d**–**f**) of silver nanoparticles obtained using alcoholic extracts of *Phoenix dactylifer*, *Acacia nilotica*, and *Ferula asafetida*. Magnification is 20,000× and the scale bar represents 100 nm for TEM images and 1 µm for SEM images.

**Figure 2:**
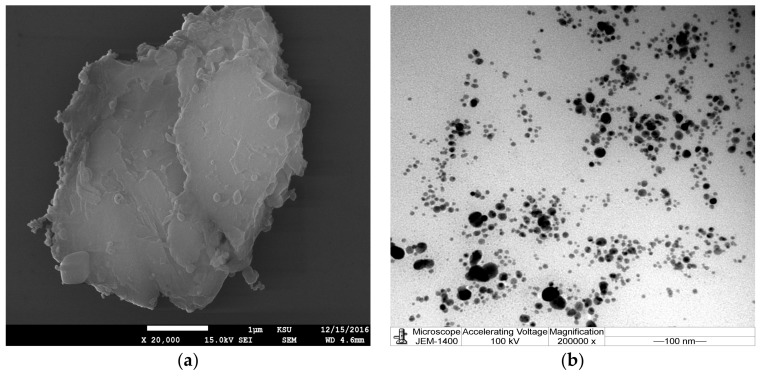

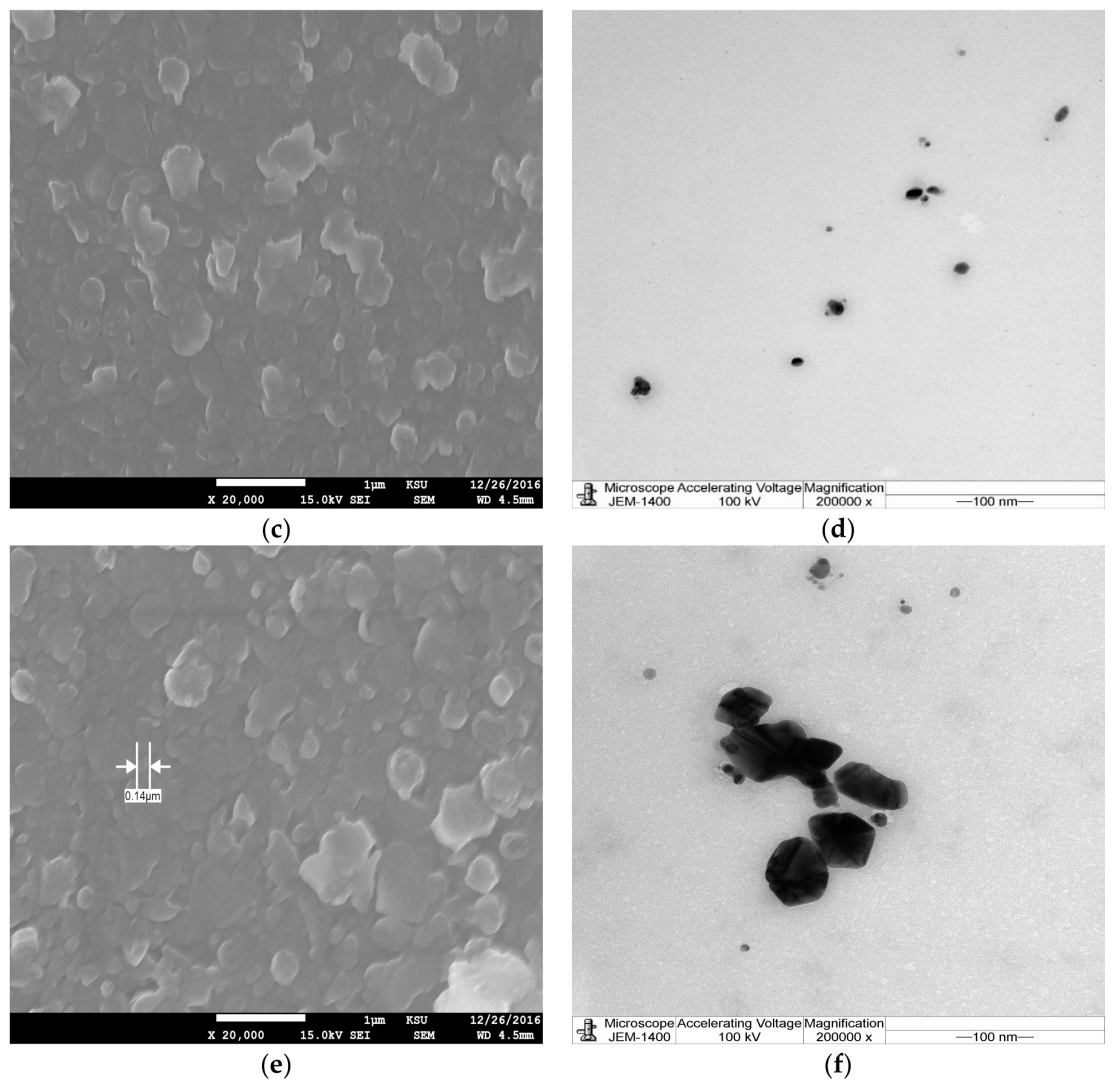
SEM images (**a**–**c**) and the corresponding TEM images (**d**–**f**) of silver nanoparticles obtained using aqueous extracts of *Phoenix dactylifer*, *Acacia nilotica*, and *Ferula asafetida*. Magnification is 20,000× and scale bar represents 100 nm for TEM images and 1 µm for SEM images.

Results from the current investigation revealed that the addition of the aqueous or ethanolic plant extract of *Phoenix dactylifera*, *Acacia nilotica*, and *Ferula asafoetida* to the silver in the form of a nitrate (0.1 mM) led to a color change of the mixture from yellowish to brown, indicating the excitation of plasmon resonance of AgNPs [3]. The color change of the mixture was time dependent, after 48 h the color was stable and appeared to be dark brown. For observing the bio-reduction process of Ag ions to AgNPs UV-vis spectroscopy was used. Absorption peaks of the mixture of AgNPs and plant extract showed silver surface plasmon resonance at 420, 425 nm, 430, 440 nm, and 430 nm for AgNPs prepared by aqueous and alcoholic extracts of *Phoenix dactylifer*, *Acacia nilotica*, and *Ferula asafetida*, respectively. SEM and TEM were used to study the AgNPs morphology, surface shape and size. Figure 1 and Figure 2 present the SEM image and corresponding TEM images for the AgNPs prepared by different plant materials in the current study. Figure 1a–c shows AgNPs obtained by using alcoholic extracts of *Phoenix dactylifer*, *Acacia nilotica*, and *Ferula asafoetida*. Figure 2a–c shows AgNPs obtained using aqueous extracts of *Phoenix dactylifer*, *Acacia nilotica*, and *Ferula asafetida.* Spherical shaped AgNPs were obtained from the *Phoenix dactylifer* ethanolic and aqueous extract using SEM and TEM; the same observations were also recorded [30] with a size of 20–60 nm. In the current study the aqueous extract of the *Phoenix dactylifer* showed an average size of 67 nm, but the ethanolic extract showed a relatively higher size (121 nm). Regarding AgNPs prepared by *Ferula asafoetida*, SEM and TEM indicated spherical shapes for both extraction methods with an average size of 105.7 and 155.7 nm for aqueous and ethanolic extracts respectively. A similar observation regarding the shape was also recorded by Sangeetha et al. [40], yet they found relatively small particle sizes (10.4 nm). Polydispersed and spherical shaped AgNPs prepared by *Acacia nilotica* with particle sizes of 100.4 and 147 nm were detected in the current study using SEM and TEM images. A range of 30–150 nm was recorded by Usha and Rachel in 2014 when they studied *A. nilotica* leaves. Furthermore, AgNPs prepared by aqueous and ethanolic extract of *Phoenix dactylifer* showed a mean zeta potential of −5.4 and −14 mV, respectively, while −35 mV was recorded by Farhadi et al. [41], and AgNPs prepared by aqueous and ethanolic extract of *F. asafoetida* showed mean values of −0.2 and −12 mV respectively. −14 and −15 mV were observed by Sangeetha et al. [40]. −0.2 and −13.2 mV were detected in the current study for AgNPs prepared by aqueous and thanolic extract of *Acacia nilotica*, respectively. AgNPs prepared by ethanolic extract showed higher a negative value than the AgNPs prepared by aqueous extract for all different plants used, indicating higher stability for the AgNPs since high negative values demonstrate the repulsion between the particles and therefore achieve stable AgNPs without accumulation [41]. Furthermore, the charge of Ag ions changing to negative may be influenced by the biomolecules from the plant extract attached to them as negative ionizable groups [42]. Negative zeta potential for AgNPs prepared by *F. asafoetida* might be related to carboxyl and hydroxyl groups of ferulic acid that capped the AgNPs surface [40]. Different plant extracts with different extraction methods showed different abilities for the preparation of AgNPs since different particle sizes and potential were observed (Table 1).

### 4.2. Fourier-Transform Infrared Spectroscopy 

The FT-IR measurements were taken for the determination of biomolecules that bound to the silver ion particles and act as reducing, capping, and stabilizing agents. The present results showed wide ranges of absorption peaks for the different plant extracts with different extraction methods used. AgNPs prepared by *Phoenix dactylifera* showed a range of 423–3310 cm^−1^, and those prepared by *Acacia nilotica* showed a range of 416–3311 cm^−1^ while those prepared by *Ferula asafoetida* showed a range of 430–3316 cm^−1^, indicating that different molecules were expected in each extract (Supplementary Materials Figure S1). Peaks at 3268–3432 cm^−1^ might indicate the stretching vibrations of H-bonded OH groups that may be found in alcohol, phenol groups, or glycosides [43]. Bands at 2158–2163 cm^−1^ might be attributed to CO hydrogen-bonded with hydroxyl groups [44]. The obtained bands from different extracts at 1632, 1633, and 1634 cm^−1^ might correspond to amide bands in proteins. Absorbance between 1600 and 1700 cm^−1^ may show the amide I vibration for proteins that could be related to C=O stretching vibration [45]. Peaks found in the current study were near 1633 cm^−1^, which was also reported for native protein indicating that the protein interacting with AgNPs did not change after binding [46]. Bands of about 1634 cm^−1^ were recorded when *Jatropha gossypifolia* was used as a bioreductant agent for AgNPs synthesis [47]. Bands at 380–580 could be related to aromatic nitrile [48]. Clear bands in the current study may suggest the presence of some organic compounds such as phenolic, glycosides, or protein in plant extracts that could be acting as stabilizing and capping agents for AgNPs. Phenolic, carboxyl and carbonyl functional groups were also detected by Farhadi et al. [41] for NPs prepared by *Phoenix dactylifera*.

### 4.3. Antibacterial Activity of Biogenic AgNPs

Biogenic AgNPs showed potent bactericidal action against tested bacterial species except AgNPs prepared by aqueous extract of *F. asafoetida* against *P. aeruginosa*. Plant aqueous extract and AgNO_3_ solution showed low antibacterial activity compared with plant alcoholic extract. The liposolubility of plant compounds could be the main reason for such activity since the bacterial cell membrane allows only for lipid soluble substances to penetrate the bacterial cell [49]. From the current study, it was clear that the ability of different plant extracts to make AgNPs from silver nitrate differs according to the plant extract and different extraction methods used; therefore, different antibacterial activity was noticed. AgNPs prepared by aqueous and alcoholic extracts mostly showed antibacterial ability against tested microbes. AgNPs prepared by *Phoenix dactylifer* and *Acacia nilotica* ethanolic extracts showed more than 50% of the antibacterial activity of Amoxicillin against *S. aureus*. AgNPs prepared by *Phoenix dactylifer* aqueous extract showed more than 85% of the Amoxicillin activity. More than 40% of Cefuroxime activity was noticed for AgNPs prepared by *Phoenix dactylifer* aqueous extract against *S. aureus*. Regarding *E. coli*, about 60%, 50%, and 30% of the activity of Amoxicillin, Cefuroxime, and Ciprofloxacin, respectively, was observed for AgNPs, but such nanoparticles were more active than Amoxicillin and Cefuroxime against *P. aeruginosa*. About 40% of the Ciprofloxacin activity was noticed for AgNPs except for those prepared by *F. asafetida*, which showed no ability against *P. aeruginosa*. Antibacterial activity of AgNPs prepared by plant extract were well documented [50,51]. On the other hand, ethanolic extract of *Phoenix dactylifer* and *Acacia nilotica* showed high antibacterial activity against all tested microbes. From the current results, it is clear that the antibacterial activity of the AgNPs that were prepared from aqueous plant extracts cannot be related to the plant extract or silver nitrate alone since the AgNPs showed a greater ability to suppress microbial growth in comparison to them. Furthermore, no special trend of inhibiting activity against gram-negative species was observed, suggesting that the AgNPs activity was not related to the structure of the bacterial cell wall.

#### 4.3.1. Tolerance Determination

An additional examination for biogenic AgNPs antibacterial potential was done at different concentration levels to determine the MIC and MBC for the tested bacterial strain. The values of the MIC and the MBC for AgNPs against all tested bacteria ranged between 12.5 to 75 µg/mL. Furthermore, the MBC/MIC ratio may reflect the susceptibility, tolerance, or resistance of the bacteria to the tested agent [52]. For all tested strains, the tolerance levels for AgNPs were two or less than two, which indicates that the AgNPs prepared by different biological materials in the current study are considered bactericidal agents [38].

#### 4.3.2. Synergistic Antimicrobial Potential of AgNPs

**Figure 3:**
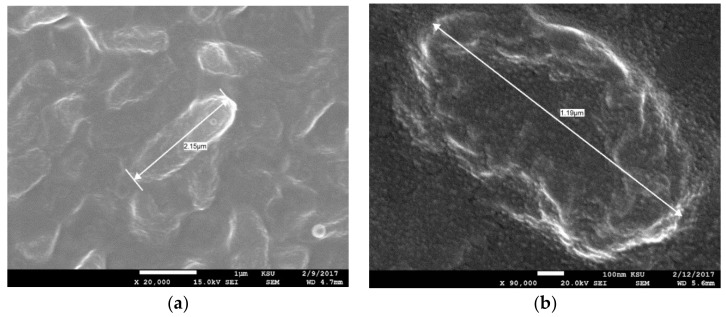
SEM micrograph of *Pseudomonas aeruginosa* loaded with AgNPs (**a**) normal cell (**b**) treated cell with AgNPs with abnormal cell wall which is also short compared to the untreated cells.

The synergistic potential of the AgNPs together with the standard antibiotics, Amoxicillin, Cefuroxime, and Ciprofloxacin, were tested against *E. coli*, *S. aureus*, and *P. aeruginosa*, with the results presented in (Table 2). Different types of antibiotics have a different mode of action against bacteria: Cefuroxime and Amoxicillin act by inhibiting bacterial cell wall synthesis, and Ciprofloxacin inhibits the enzyme bacterial DNA gyrase and prevents bacterial DNA replication during bacterial growth and reproduction [53]. Both antibiotics and AgNPs were mixed at their low concentration and their activities were evaluated. The effect was observed against tested bacteria, but *P. aeruginosa* showed resistance when treated with AgNPs mixed with Amoxicillin and some AgNPs mixed with Cefuroxime. Mixing AgNPs with Ciprofloxacin showed strong positive antibacterial activity against all tested microbes. [53,54,55,56] suggested that a better interaction of the mixture with the pathogen due to the bonding reaction may lead to a positive synergistic effect of AgNPs-antibiotics mixture when some MDR bacteria were studied. Active uptake of the AgNPs-ciprofloxacin mixture by bacteria could be the reason for the cells damage since the ciprofloxacin inhibits the enzyme bacterial DNA gyrase and prevents DNA replication [53]. Generally, synergistic abilities of AgNPs with the antibiotics may reduce the use of antibiotics and therefore, reduce the development of antibiotic resistant microbes. In the current study, no special trends or observations were recorded when the synergistic impact of antibiotics in combination with AgNPs were investigated against tested bacteria [53]. Furthermore, different biogenic AgNPs from different backgrounds showed different synergistic effects against different tested strains. On the other hand, the SEM micrograph for *Pseudomonas* sp. showed a relative change in the bacterial membrane morphology when treated bacteria was tested but also the cell was short compared with a normal cell (Figure 3). The mechanism for AgNPs against bacteria is not absolutely understood, but [28] Das et al. assumed that morphological changes in the bacterial membrane may affect the permeability and therefore cell death may appear.

### 4.4. Cell Viability Study

**Figure 4:**
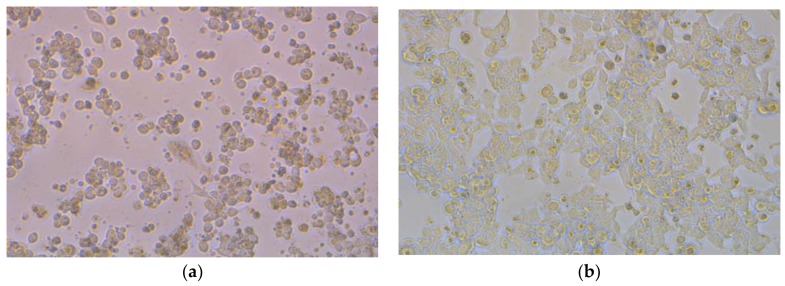
Morphological changes in LoVo cells treated with 46.15 μg/mL AgNPs mediated by *Ferula asafetida* extracts for 72 h (**b**); (**a**) is the control. Images were captured by a phase contrast microscope (Leica, Germany), (Magnification: 200×).

The cytotoxic potency of AgNPs mediated by a plant extract was tested against human colon cancer LoVo cell lines by the MTT test. There was no activity for AgNPs prepared by aqueous plants extract against tested cells, but AgNPs prepared by ethanolic plant extract showed a clear cytotoxic effect. 46.15 ± 2.0, 58.02 ± 2.1, and 69.73 ± 2.02 μg/mL are the IC50 values for AgNPs mediated by *Ferula asafetida*, *Acacia nilotica*, and *Phoenix dactylifer*, respectively. The silver nanoparticles showed good anticancer activity against LoVo cells line with IC50 values lower than that as reported by [57], and they reported against HCT-116 Colon Cancer Cells when *Commelina nudiflora* L. Aqueous Extract was used for the biogenic synthesis of AgNPs [58]. The antitumor ability of *asafoetida* on breast cancer is well-documented [59]. Current investigations show significant antiproliferative action of biosynthesized AgNPs by alcoholic plant extract against LoVo cell lines that may lead to growth cell suppression. Such an ability could be related to the synergetic effect of the biosynthesized nanoparticles and the bioactive molecules attached to their surface [57]. Furthermore, it could be assumed that active secondary metabolites that have an antitumor effect were more effective when dissolved in alcohol than in water. In anticancer treatment research, apoptosis as a biological tool that destroys the abnormal cells is considered a smart screening procedure [60]. Generally, a series of modifications appear as a sign of apoptosis or morphologically programmed cell deaths such as chromatin and cytoplasm condensation [61]. In the current study, a phase contrast microscope was used for morphological observations; normally developed cells in the control group that adhered to the bottom of the plate with round cell nuclei were noticed. Following treatment with IC50 concentrations of AgNPs mediated by ethanolic plant extract for 72 h, apoptosis-like symptoms appeared in the cell such as cytoplasmic condensation and detachment from the neighboring cell, swelling of the cell, and a rounded shape. Generally, an increase in the number of apoptosis-like bodies in the tested LoVo cells was observed (Figure 4). Moreover, Buttacavoli et al. [62] detected a similar trend in their observations when they studied the bio-generated silver nanoparticles against human breast cancer cell lines. Generally, cellular morphological changes in the cell might be due to the disturbance in cell composition due to the cell surface and AgNPs interaction [63].

Based on similar studies, our current investigation has generated interesting findings that can be expressed in useful applications. Such advantages include the success of our plant extracts in the rapid formation of small AgNPs (67.8–155.7 nm) from AgNO_3_, the prepared NPs exhibited antimicrobial activity against *E. coli*, *S. aureus*, and *P. aeruginosa* close to that of commercial antibiotics and in some cases even greater. Also, the prepared NPs via our plant extract possessed cytotoxicity against LoVo cell lines; therefore, the plant extracts used in synthesizing AgNPs could be considered as an added value in the development of nano-medicine and pharmaceutical production for the treatment of cancer cells and pathogenic microorganisms.

## 5. Conclusions

Antibiotics and anticancer chemotherapeutic drugs are very costly and toxic, besides being a drug resistance problem. Consequently, looking for alternative medicines is essential. Silver nanoparticles produced with an eco-friendly approach is efficient, boasts limited side effects, and less cost. In the current investigation, the aqueous and alcoholic extract of *Ferula asafetida*, *Acacia nilotica*, and *Phoenix dactylifer* showed the ability to produce AgNPs from AgNO_3_; such particles had an ability to suppress the growth of some pathogenic microorganisms and colon cancer cells. Therefore, this AgNP should be seriously considered as an antibiotic and anticancer drug. Our investigations in the future will focus mainly on answering specific questions and the knowledge gained will enhance our understanding of the machinery of the nanoparticles and will integrate the picture for biological and biotechnological applications.

## Figures and Tables

**Table 1:** Average particle size (nm), Average Zeta potential (mV) of biogenic synthesized silver nanoparticles (AgNPs) and their Zone of inhibition (mm) against *Escherichia coli*, *Staphylococcus aureus*, and *Pseudomonas aeruginosa*.

Treatment	Average Particle Size (nm)	Average Zeta Potential (mV)	Inhibition Zone + Gram (mm)	Inhibition Zone − Gram (mm)	
			*S. aureus*	*E. coli*	*P. aeruginosa*
AKN	121.3 ± 1.1	−14 ± 1	14.5 ± 1.5	15.8 ± 0.8	14.0 ± 0.6
AWN	67.8 ± 0.3	−5.4 ± 0.3	17.8 ± 1.3	11.8 ± 1.0	15.1 ± 0.8
GKN	147 ± 2.5	−13.2 ± 0.2	13.4 ± 2	19.6 ± 2.0	14.10.5
GWN	100.4 ± 0.9	−0.2 ± 0.3	11.2 ± 0.1	12.0 ± 1.4	15.8 ± 0.1
HKN	155.7 ± 1.5	−12 ± 0.7	11.8 ± 1.6	10.3 ± 1.1	14.6 ± 2.6
HWN	105.7 ± 0.6	−0.2 ± 0.6	10.0 ± 0.5	10.7 ± 2.0	0
AK	-	-	32.2 ± 0.1	18 ± 0.3	18.8 ± 0.5
AW	-	-	23.4 ± 2.4	0	14.1 ± 0.4
GK	-	-	21.2 ± 2	19.6 ± 2	14.1 ± 0.5
GW	-	-	10.6 ± 1.2	0	0
HK	-	-	0	0	0
HW	-	-	0	0	0
Ag ions	-	-	1.3	1.5	1
Amoxicillin (AMO)	-	-	22.9 ± 0.5	17.9 ± 0.7	0
Cefuroxime (CXM)	-	-	29.4 ± 1.0	26.9 ± 0.5	0
Ciprofloxacin (CIP)	-	-	27 ± 1.9	35 ± 2.0	37 ± 0.6

Data expressed as mean ± SD. AKN = AgNPs prepared by alcoholic extract of *Phoenix dactylifer*, AWN *=* AgNPs prepared by aqueous extract of *Phoenix dactylifer*, GKN *=* AgNPs prepared by alcoholic extract of *Acacia nilotica*, GWN *=* AgNPs prepared by aqueous ethanolic extract of *Acacia nilotica*, HKN *=* AgNPs prepared by alcoholic extract of *Ferula asafoetida*, HWN *=* AgNPs prepared by aqueous extract of *Ferula asafetida.* AK, GK and HK *=* alcoholic extract of *Phoenix dactylifer*, *Acacia nilotica* and *Ferula asafetida* respectively and AW, GW and HW = aqueous extract of *Phoenix dactylifer*, *Acacia nilotica* and *Ferula asafetida* respectively.

**Table 2:** Zone of inhibition, mm as an effect of different antibiotics (Amoxicillin (AMOX), Cefuroxime (CEF) and Ciprofloxacin (CIP)) in combination with AgNPs at MIC (50 mg/L) against *Escherichia coli*, *Staphylococcus aureus* and *Pseudomonas aeruginosa*.

Treatment	Microbes	AMOX	CEF	CIP
ATB + 1	*E. coli*	0	25 ± 1	27.7 ± 0.5
	*S. aureus*	16 ± 0.5	33.3 ± 1.5	24.7 ± 0.5
	*P. aeruginosa*	0	7.7 ± 1.5	36.3 ± 1.7
ATB + 2	*E. coli*	8.3 ± 0.5	21.8 ± 0.5	28 ± 1
	*S. aureus*	18.3 ± 0.5	31.3 ± 2.5	24.7 ± 0.5
	*P. aeruginosa*	0	0	32.7 ± 1.2
ATB + 3	*E. coli*	0	24.3 ± 0.5	26.7 ± 0.5
	*S. aureus*	15.3 ± 2.1	26 ± 1	23.7 ± 0.5
	*P. aeruginosa*	0	0	31.7 ± 1.5
ATB + 4	*E. coli*	7.3 ± 0.5	23 ± 0	24 ± 0
	*S. aureus*	16.7 ± 0.5	28.3 ± 0.5	22.3 ± 0.5
	*P. aeruginosa*	0	0	30.3 ± 0.5
ATB + 5	*E. coli*	0	22 ± 1	34 ± 0
	*S. aureus*	15.7 ± 0.5	28.7 ± 1.5	28 ± 1
	*P. aeruginosa*	0	8 ± 1.7	30.7 ± 2
ATB + 6	*E. coli*	9.3 ± 0.5	29.3 ± 0.5	26.3 ± 0.5
	*S. aureus*	18 ± 1	30 ± 0	26.3 ± 1.5
	*P. aeruginosa*	0	0	25.7 ± 0.5

Data expressed as mean ± SD. 1 = AgNPs prepared by aqueous extract of *Ferula asafetida*, 2 *=* prepared by ethanolic extract of *Ferula asafetida*, 3 *=* prepared by aqueous extract of *Acacia nilotica*, 4 *=* prepared by aqueous ethanolic extract of *Acacia nilotica*, 5 *=* prepared by aqueous extract of *Phoenix dactylifer*, 6 *=* prepared by aqueous ethanolic extract of *Phoenix dactylifer.*

## Supplementary Materials

The following are available online at http://www.mdpi.com/2079-4991/8/6/382/s1, Figure S1: FTIR spectra of synthesized silver nanoparticles by the alcoholic and aqueous extract of *Phoenix dactylifer, Acacia nilotica* and *Ferula asafetida*.
