# Supplementary material for: Antibacterial and Cytotoxic Potential of Biosynthesized Silver Nanoparticles by Some Plant Extracts
Source: Nanomaterials (Basel). 2018 May 30;8(6):382. doi: 10.3390/nano8060382 (PMC6027515; doi:10.3390/nano8060382)
Supplement: Supplementary file 1 [file nanomaterials-08-00382-s001.pdf]

# Supplementary Nanomaterials:

# Antibacterial and Cytotoxic Potential of Biosynthesized Silver Nanoparticles by Some Plant Extracts

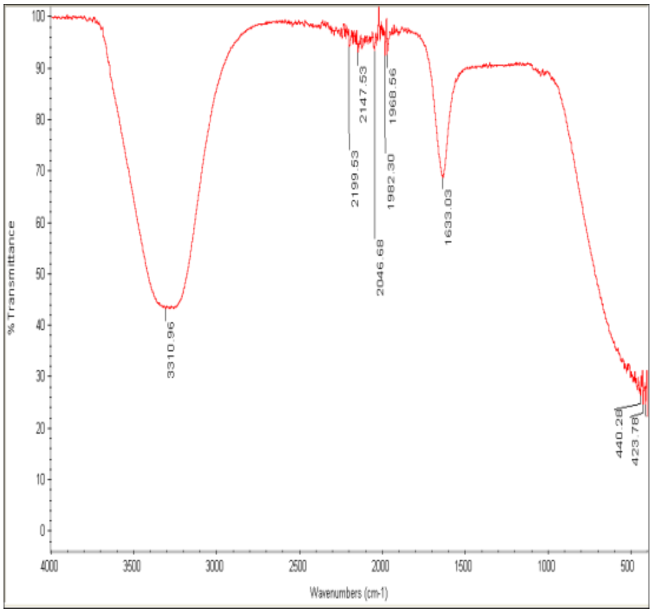

FTIR spectra synthesized silver nanoparticle by *Phoenix dactylifer* alcoholic extract

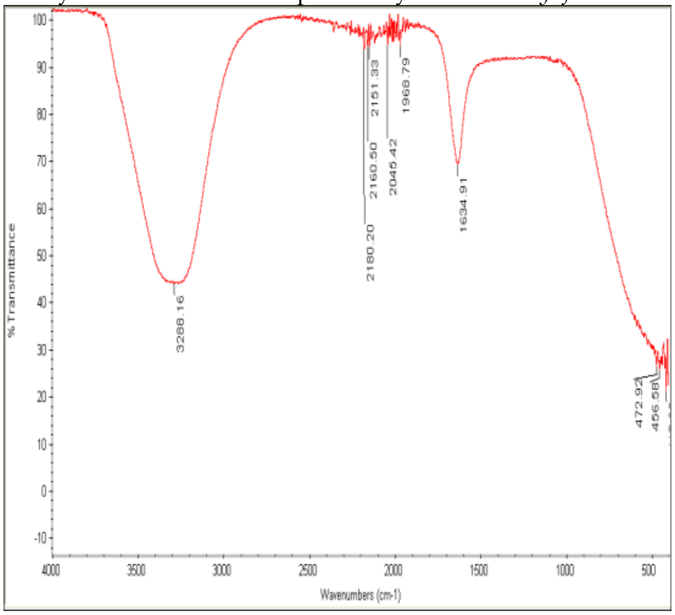

FTIR spectra synthesized silver nanoparticle by *Phoenix dactylifer* aqueous extract

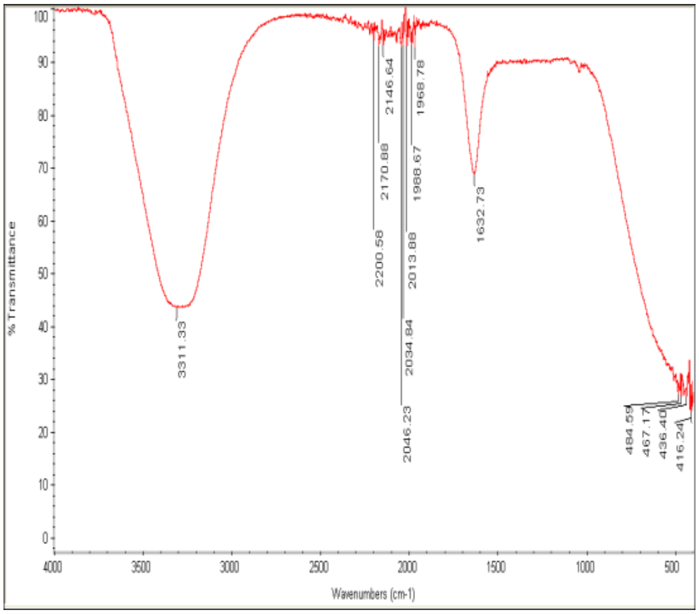


FTIR spectra synthesized silver nanoparticle by *Acacia nilotica* alcoholic extract

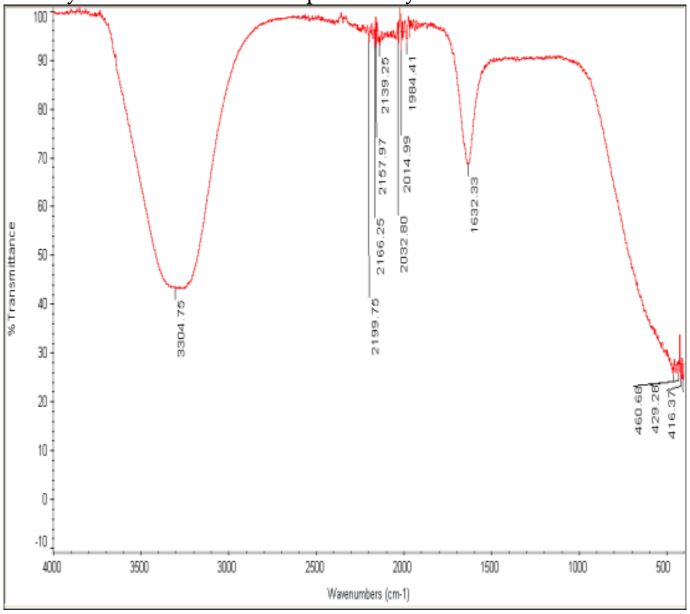


FTIR spectra synthesized silver nanoparticle by *Acacia nilotica* aqueous extract

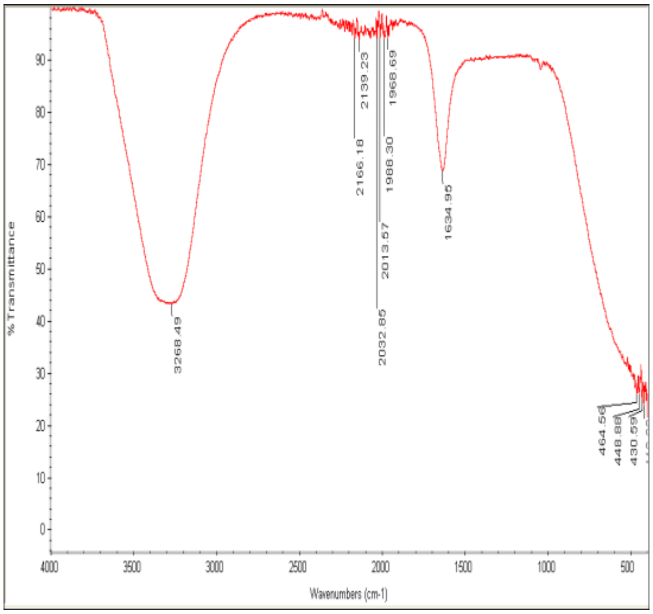


FTIR spectra synthesized silver nanoparticle by *Ferula asafetida* alcoholic extract

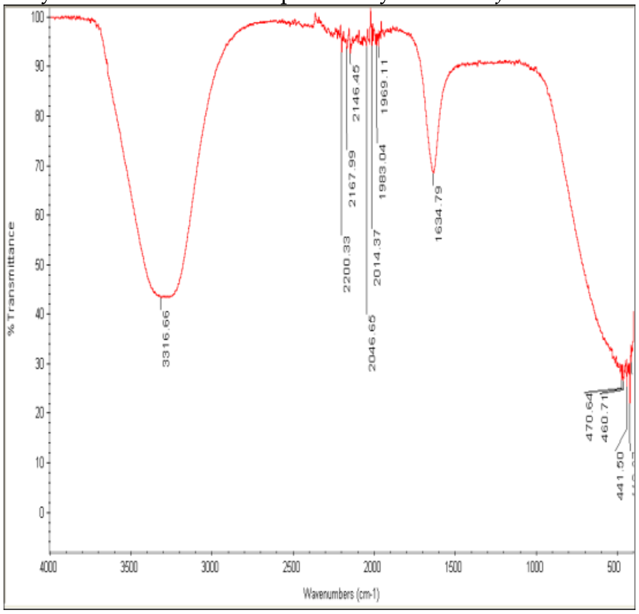


FTIR spectra synthesized silver nanoparticle by *Ferula asafetida* aqueous extract

Figure S1: FTIR spectra of synthesized silver nanoparticles by the alcoholic and aqueous extract of *Phoenix dactylifer, Acacia nilotica* and *Ferula asafetida*.
